# Editorial for Special Issue on Herbal Medicines and Natural Products

**DOI:** 10.3390/medicines2040328

**Published:** 2015-11-16

**Authors:** Zhi-Wei Zhou, Shu-Feng Zhou

**Affiliations:** Department of Pharmaceutical Sciences, College of Pharmacy, University of South Florida, Tampa, FL 33612, USA; E-Mail: zzhou1@health.usf.edu

**Keywords:** herbal medicines, natural products, drug development, pharmacovigilance

## Abstract

Herbal medicines and natural products have been the most productive source of drug development and there is a large line of evidence on the applications of herbal medicines and natural products for the management of body function and the treatment of aliments. The multiple bioactive components in herbal medicines and natural products can explain the multiple targets effect in their medical applications. The increasing usage of state-of-art computational, molecular biological, and analytical chemistry techniques will promote the exploration of the pharmacological effect of previously inaccessible sources of herbal medicines and natural products. Notably, with the increasing reports on the safety issues regarding the medical use of herbal medicines and natural products, the awareness of pharmacovigilance in herbal medicines and natural products needs to be strengthened. To prevent the adverse drug reactions related to herbal medicines and natural products, physicians need to be aware of potential risks and alert patients in the use of herbal medicines and natural products.

There is a wealth of evidence on the application of herbal medicines and natural products for the management of body functions and the treatment of a variety of ailments, owning to the diversity of bioactive components with various pharmacological effects [1]. Also, herbal medicines and natural products have been the most productive source of lead compounds for the development of drugs. There are over 100 compounds/products that are in clinical development or use. They are mainly used in anti-cancer and anti-infective areas, and to a lesser extent, neuropharmacological, cardiovascular/gastrointestinal, inflammation, metabolic, skin, hormonal, and immunosuppressant fields [2]. Of note, artemisinin is a classical antimalarial drug extracted and isolated from wormwood plant *Artemisia annua* by Chinese scientists in 1972 and the discoverers of artemisinin were awarded the Nobel Prize in 2015. Recently, application of state-of-art techniques has been increasing the availability of novel compounds, including primary and secondary metabolites (SM), which can be conveniently produced in bacteria, yeasts, or plants; and combinatorial chemistry and computational approaches are being based on herbal medicine and natural product scaffolds to create screening libraries that closely resemble drug-like compounds. The current special issue covers the modes of actions of herbal medicines and SM, the potential risks of herbal medicines and natural products, and natural products based drug discovery and development.

Wink [3] delineated the modes of action of herbal medicines and plant SM for medicinal usage. SMs exert a broad spectrum of biological and pharmacological activities and can be used for the treatment of infections, health disorders or diseases. Wink’s paper [3] showed that SM interacted with macromolecules, the main targets in cells, such as proteins, biomembranes or nucleic acids. SM can form hydrogen, hydrophobic and ionic bonds with their targets, consequently, modulating their 3D structures and bioactivities, such as inhibition of enzymatic activity and disruption of ligand-receptor binding. Also, the paper pointed out that the multi-target activities of SM provided an explanation for their medical application for various ailments which involve several targets.

Want *et al.* [4] focused on the effect of commonly used dietary supplements on coagulation function during surgery. His paper provided a systematic view on the effect of dietary supplements on coagulation and platelet function, including 11 herbal medicines (echinacea, ephedra, garlic, ginger, ginkgo, ginseng, green tea, kava, saw palmetto, St John’s wort, and valerian) and four other dietary supplements (coenzyme Q10, glucosamine and chondroitin sulfate, fish oil, and vitamins). The paper showed that there were bleeding risks of garlic, ginkgo, ginseng, green tea, saw palmetto, St John’s wort, and fish oil and that there was cardiovascular instability of ephedra, ginseng, and kava. Also, dietary supplements interacted with therapeutics in pharmacodynamics and pharmacokinetics in the perioperative period. Therefore, it is of clinical importance for physicians to familiarize the perioperative effects of commonly used dietary supplements and it is prudent to advise their discontinuation before surgery, to prevent potential risks in coagulation and platelet function.

Ullah [5] discussed sulforaphone (SFN), an isothiocyanate in a cancer chemoprevention paradigm and provided the current understanding of the cancer chemopreventive pharmacology of SFN towards its anticancer potential. The paper showed that SFN was considered to be a potent anti-cancer agent, due to its favorable properties. SFN exhibits a good bioavailability as it can reach high plasma and intracellular concentrations. SFN inhibits histone deacetylase (HDAC) activity. SFN shows more potent cancer cell killing effect than that towards normal cells. Ullah’s paper [5] also showed that SFN kills cancer cell may be via the regulation of nuclear factor erythroid 2-related factor 2 mediated redox homeostasis signaling pathway, inhibition of HDAC, induction of cell cycle arrest, promotion of apoptosis, and modulation of transcription factors. The emerging evidence from epidemiological and experimental studies enhances the clinical plausibility and translational value of SFN in cancer chemoprevention.

Findeis *et al*. [6] highlighted the natural products and natural product-derived γ secretase modulators from *Actaea racemosa* extracts in the treatment of Alzheimer’s disease (AD). AD is characterized by pathogenic oligomerization, aggregation, and deposition of amyloid β peptide (Aβ), resulting in severe neuronal toxicity and associated cognitive dysfunction. In particular, accumulation of Aβ42 is associated with increased cellular toxicity and rapidity of disease progression. Therefore, the strategy to reduce the level of Aβ42 by modulating the proteolytic activity of the γ secretase complex has been undertaken. Findeis’ paper [6] discussed a program that sought to develop such γ secretase modulators based on novel natural products identified in the extract of *A. racemosa*, the well-known botanical black cohosh. SPI-014, the bioactive component, has been extensively studied in terms of structure activity relationship, which inspired the replacement of both the sugar and acetate moieties with more stable alternatives, improving the drug-like properties and resulting in the development of SPI-1865.

In conclusion, there are a number of promising drug candidates in the current development pipeline that are originated from herbal medicines or natural products, which can spur the exploration of the biological activities of previously inaccessible sources of herbal medicines and natural products with the application of sophisticate computational, molecular biological, and analytical chemistry approaches. However, with the increasing use of herbal medicines and natural products, the pharmacovigilance of herbal medicines and natural products is becoming more pressing; and it is of great importance to monitor the safety of herbal medicines and natural products in medical application.
